# Real-World Gait Detection Using a Wrist-Worn Inertial Sensor: Validation Study

**DOI:** 10.2196/50035

**Published:** 2024-05-01

**Authors:** Felix Kluge, Yonatan E Brand, M Encarna Micó-Amigo, Stefano Bertuletti, Ilaria D'Ascanio, Eran Gazit, Tecla Bonci, Cameron Kirk, Arne Küderle, Luca Palmerini, Anisoara Paraschiv-Ionescu, Francesca Salis, Abolfazl Soltani, Martin Ullrich, Lisa Alcock, Kamiar Aminian, Clemens Becker, Philip Brown, Joren Buekers, Anne-Elie Carsin, Marco Caruso, Brian Caulfield, Andrea Cereatti, Lorenzo Chiari, Carlos Echevarria, Bjoern Eskofier, Jordi Evers, Judith Garcia-Aymerich, Tilo Hache, Clint Hansen, Jeffrey M Hausdorff, Hugo Hiden, Emily Hume, Alison Keogh, Sarah Koch, Walter Maetzler, Dimitrios Megaritis, Martijn Niessen, Or Perlman, Lars Schwickert, Kirsty Scott, Basil Sharrack, David Singleton, Beatrix Vereijken, Ioannis Vogiatzis, Alison Yarnall, Lynn Rochester, Claudia Mazzà, Silvia Del Din, Arne Mueller

**Affiliations:** 1 Novartis Biomedical Research Novartis Pharma AG Basel Switzerland; 2 Department of Biomedical Engineering Tel Aviv University Tel Aviv Israel; 3 Translational and Clinical Research Institute Faculty of Medical Sciences Newcastle University Newcastle upon Tyne United Kingdom; 4 Department of Electronics and Telecommunications Politecnico di Torino Turin Italy; 5 Department of Electrical, Electronic and Information Engineering University of Bologna Bologna Italy; 6 Center for the Study of Movement, Cognition and Mobility Neurological Institute Tel Aviv Sourasky Medical Center Tel Aviv Israel; 7 Department of Mechanical Engineering and Insigneo Institute for In Silico Medicine The University of Sheffield Sheffield United Kingdom; 8 Machine Learning and Data Analytics Lab Department of Artificial Intelligence in Biomedical Engineering Friedrich-Alexander-Universität Erlangen-Nürnberg Erlangen Germany; 9 Health Sciences and Technologies-Interdepartmental Center for Industrial Research (CIRI-SDV) University of Bologna Bologna Italy; 10 Laboratory of Movement Analysis and Measurement Ecole Polytechnique Federale de Lausanne Lausanne Switzerland; 11 National Institute for Health and Care Research (NIHR) Newcastle Biomedical Research Centre (BRC) Newcastle University and The Newcastle upon Tyne Hospitals NHS Foundation Trust Newcastle upon Tyne United Kingdom; 12 Robert Bosch Gesellschaft für Medizinische Forschung Stuttgart Germany; 13 Unit Digitale Geriatrie Universitätsklinikum Heidelberg Heidelberg Germany; 14 The Newcastle upon Tyne Hospitals NHS Foundation Trust Newcastle upon Tyne United Kingdom; 15 Barcelona Institute for Global Health (ISGlobal) Barcelona Spain; 16 Universitat Pompeu Fabra Barcelona Spain; 17 CIBER Epidemiología y Salud Pública (CIBERESP) Madrid Spain; 18 Insight Centre for Data Analytics University College Dublin Dublin Ireland; 19 School of Public Health, Physiotherapy and Sports Science University College Dublin Dublin Ireland; 20 Newcastle upon Tyne Hospitals NHS Foundation Trust Newcastle upon Tyne United Kingdom; 21 McRoberts BV The Hague Netherlands; 22 Department of Neurology University Medical Center Schleswig-Holstein Campus Kiel Kiel Germany; 23 Sagol School of Neuroscience Tel Aviv University Tel Aviv Israel; 24 Department of Physical Therapy Faculty of Medical & Health Sciences Tel Aviv University Tel Aviv Israel; 25 Rush Alzheimer’s Disease Center Rush University Medical Center Chicago, IL United States; 26 Department of Orthopaedic Surgery Rush Medical College Chicago, IL United States; 27 Department of Sport, Exercise and Rehabilitation Northumbria University Newcastle Newcastle upon Tyne United Kingdom; 28 Department of Neuroscience The University of Sheffield Sheffield United Kingdom; 29 Sheffield NIHR Translational Neuroscience BRC Sheffield Teaching Hospitals NHS Foundation Trust Sheffield United Kingdom; 30 Department of Neuromedicine and Movement Science Norwegian University of Science and Technology Trondheim Norway

**Keywords:** digital mobility outcomes, validation, wearable sensor, walking, digital health, inertial measurement unit, accelerometer, Mobilise-D

## Abstract

**Background:**

Wrist-worn inertial sensors are used in digital health for evaluating mobility in real-world environments. Preceding the estimation of spatiotemporal gait parameters within long-term recordings, gait detection is an important step to identify regions of interest where gait occurs, which requires robust algorithms due to the complexity of arm movements. While algorithms exist for other sensor positions, a comparative validation of algorithms applied to the wrist position on real-world data sets across different disease populations is missing. Furthermore, gait detection performance differences between the wrist and lower back position have not yet been explored but could yield valuable information regarding sensor position choice in clinical studies.

**Objective:**

The aim of this study was to validate gait sequence (GS) detection algorithms developed for the wrist position against reference data acquired in a real-world context. In addition, this study aimed to compare the performance of algorithms applied to the wrist position to those applied to lower back–worn inertial sensors.

**Methods:**

Participants with Parkinson disease, multiple sclerosis, proximal femoral fracture (hip fracture recovery), chronic obstructive pulmonary disease, and congestive heart failure and healthy older adults (N=83) were monitored for 2.5 hours in the real-world using inertial sensors on the wrist, lower back, and feet including pressure insoles and infrared distance sensors as reference. In total, 10 algorithms for wrist-based gait detection were validated against a multisensor reference system and compared to gait detection performance using lower back–worn inertial sensors.

**Results:**

The best-performing GS detection algorithm for the wrist showed a mean (per disease group) sensitivity ranging between 0.55 (SD 0.29) and 0.81 (SD 0.09) and a mean (per disease group) specificity ranging between 0.95 (SD 0.06) and 0.98 (SD 0.02). The mean relative absolute error of estimated walking time ranged between 8.9% (SD 7.1%) and 32.7% (SD 19.2%) per disease group for this algorithm as compared to the reference system. Gait detection performance from the best algorithm applied to the wrist inertial sensors was lower than for the best algorithms applied to the lower back, which yielded mean sensitivity between 0.71 (SD 0.12) and 0.91 (SD 0.04), mean specificity between 0.96 (SD 0.03) and 0.99 (SD 0.01), and a mean relative absolute error of estimated walking time between 6.3% (SD 5.4%) and 23.5% (SD 13%). Performance was lower in disease groups with major gait impairments (eg, patients recovering from hip fracture) and for patients using bilateral walking aids.

**Conclusions:**

Algorithms applied to the wrist position can detect GSs with high performance in real-world environments. Those periods of interest in real-world recordings can facilitate gait parameter extraction and allow the quantification of gait duration distribution in everyday life. Our findings allow taking informed decisions on alternative positions for gait recording in clinical studies and public health.

**Trial Registration:**

ISRCTN Registry 12246987; https://www.isrctn.com/ISRCTN12246987

**International Registered Report Identifier (IRRID):**

RR2-10.1136/bmjopen-2021-050785

## Introduction

Digital mobility outcomes (DMOs) such as walking speed show promise for assessing and predicting clinical outcomes in various medical conditions [1-4]. However, the traditional assessment of gait characteristics in clinical environments is often limited by infrequent, short-duration assessments and artificial measurement conditions [5,6]. Thus, the goal of ongoing research is to transfer gait assessment into the real-world to assess a patient’s everyday walking performance, investigate treatment and medication effects, and monitor fluctuating disease symptoms over long and continuous periods [7].

Typically, waist or lower limb–worn inertial sensors including accelerometers and gyroscopes are used to assess gait impairment, and numerous studies present implementation and validation of respective algorithms [8-12]. However, wrist-worn inertial sensors might be more acceptable to participants than lower back sensors and thus better suitable for large-scale studies over prolonged periods and are largely available due to the advent of smartwatches and fitness trackers [13,14].

Traditionally, wrist-worn sensors have been used to detect everyday life activities, estimate step counts, and quantify time spent in different physical activity levels [13,15,16]. Even though actigraphy allows real-world activity to be assessed as part of mobility, it might not deliver accurate insight into gait impairment as assessed by spatiotemporal gait parameters. The relevance of investigating real-world gait performance in more detail has been highlighted by recent research [5]. Accordingly, there is also a rising interest in the use of wrist-worn sensors for gait assessment in the real world, ranging from gait and stride detection [17-20] to the estimation of spatiotemporal gait parameters [21,22].

The real-world measurement paradigm promises new insights into everyday movement abilities. Large amounts of data may better represent a patient’s everyday behavior and capture rare but important episodes. An important first step toward assessing gait in real-world settings is the identification of continuous gait sequences (GSs). Those sequences can serve as preselected regions of interest containing gait in long, continuous recordings before more computationally complex algorithms for DMO extraction are applied [23]. Furthermore, the focus on GSs reduces the risk of estimating nonmeaningful DMOs in nongait conditions. Finally, extracted GSs and their duration can potentially differentiate between disease-related and healthy walking behavior [24,25].

Accurate gait sequence detection (GSD) using wrist-worn inertial sensors is, however, challenging due to several reasons. First, compared to other sensor locations, the complexity of arm movements is challenging for the extraction of mobility in general and gait parameters in particular [15]. Upper limbs are complex locations to assess DMOs due to the high movement variability and individual preferences of the amount of arm swing. Second, the use of upper limbs for a wide variety of functions other than gait, movement constraints due to walking with the hands in the pockets or holding a bag or other dual-task walking, upper limb injuries, and walking aid use may confound the data. Finally, validation data sets that include both wrist and reference data for the assessment of real-world concurrent validity in multiple disease conditions have not been available so far. Validation studies with reference data have mostly been restricted to healthy adults [22,26-29].

Various approaches for gait detection also from the wrist position have been proposed [17,21,30,31], and the aim of this study was to identify, compare, and rank available state-of-the-art algorithms for GSD based on wrist-worn inertial sensors using labeled real-world data from diverse disease and healthy groups from the Mobilise-D technical validation study [32]. In addition, the wrist-worn sensor results were compared to the outcomes generated from the best-performing algorithms for the lower back inertial sensor to allow conclusions about GSD accuracy between different sensor positions.

The results of this study can help decision makers in clinical studies and possibly in public health to recommend the use of either wrist or lower back–worn inertial sensors. This could allow for more agnostic data collection protocols to be adopted. Patients will benefit as this technology will facilitate the assessment of gait impairment in real-world conditions that may allow quantifying a meaningful aspect of life.

## Methods

### Ethical Considerations

Ethics approval was obtained at the individual sites (London-Bloomsbury Research Ethics Committee, 19/LO/1507; Helsinki Committee, Tel Aviv Sourasky Medical Center, Tel Aviv, Israel, 0551-19TLV; ethical committee of the medical faculty of The University of Tübingen, 647/2019BO2; and ethical committee of the medical faculty of Kiel University, D438/18; University of Sheffield Research Ethics Committee, 029143). All participants provided written informed consent before participating. The analysis is based on pseudonymized data, and anonymized data will be published by the Mobilise-D consortium. Participants in this study were not compensated.

### Participants

#### Overview

For optimizing and evaluating algorithms for GSD, 2 separate data sets from the Mobilise-D technical validation study were used. This multicentric observational study with the aim of validating real-world DMOs included different patient and healthy populations. The study’s experimental protocol including all inclusion and exclusion criteria have previously been described in more detail in [32].

#### Optimization Sample

To optimize algorithms for wrist position, including parameter tuning, a separate optimization data set was used. This data set was obtained during a test run within the Mobilise-D project, distinct from the validation study. As a result, it exclusively included healthy participants. Real-world gait data of 11 young and healthy adults were assessed (Sheffield Teaching Hospitals NHS Foundation Trust and University of Sassari, Italy) as part of the Mobilise-D technical validation study. They were asked to follow the same experimental protocol as the validation data set.

#### Validation Sample

A convenience sample of 108 participants across 5 different disease groups and 1 control group with healthy older adults (HAs) were recruited. The data of those participants served as *validation data set* for the final evaluation of algorithm performance. The participant groups included patients with chronic obstructive pulmonary disease, Parkinson disease, multiple sclerosis (MS), proximal femoral fracture (PFF; hip fracture recovery), and congestive heart failure (CHF). Recruitment was performed at 5 sites: the Newcastle upon Tyne Hospitals NHS Foundation Trust, United Kingdom; Sheffield Teaching Hospitals NHS Foundation Trust, United Kingdom (London-Bloomsbury Research Ethics Committee, 19/LO/1507); Tel Aviv Sourasky Medical Center, Israel (Helsinki Committee, Tel Aviv Sourasky Medical Center, Tel Aviv, Israel, 0551-19TLV); Robert Bosch Foundation for Medical Research, Germany (ethical committee of the medical faculty of the University of Tübingen, 647/2019BO2); and University of Kiel, Germany (ethical committee of the medical faculty of Kiel University, D438/18).

### Protocol

Activities of the participants were assessed during 2.5 hours of real-world living undergoing their normal activities (home or work or community or outdoor). They were also asked to perform a limited number of predefined activities (outdoor walking, walking up and down a slope and stairs, and moving from one room to another), if they felt comfortable to do so [33].

The participants were equipped with an inertial sensor worn at the wrist on the nondominant hand (target sensor from which our analysis data are derived) and a validated multisensor system, the INDIP (inertial module with distance sensors and pressure insoles) as reference [32,34]. In particular, the INDIP system included 2-feet inertial sensors attached to the shoelaces with clips (instep position), 2 distance sensors positioned asymmetrically with Velcro over the ankles, and 2 pressure insoles. GSD from the reference INDIP system has previously been described [34]. Furthermore, the INDIP system has been validated across the same patient and healthy adult groups showing excellent results and reliability in the qualification of mobility outcomes in laboratory and free-living environments [34-38]. The decision to place the sensor on the nondominant hand balances participant comfort, practicality, and data quality as it minimized interference with other daily tasks (such as writing, typing, and handling objects) and ensured consistent data collection.

Lower back data were collected by a McRoberts Dynaport MoveMonitor wearable inertial sensor (sampling frequency: 100 Hz, triaxial acceleration range: ±8g or resolution: 1 mg, triaxial gyroscope range: ±2000 dps or resolution: 70 mdps), which was attached to the lower back (L5) with an elastic belt and Velcro fastening. The INDIP system, the wrist inertial sensor (identical to those incorporated in the INDIP), and the lower back MR device were synchronized using their timestamp (±10 ms) and stored in a standardized and integrated data structure [39].

### Selection and Optimization of Gait Detection Algorithms

We identified algorithms from the literature potentially suitable for gait detection from lower back and wrist-worn inertial sensors. Our algorithm selection was based on previous work for gait detection from the lower back [11] and the availability of code of the algorithm. Furthermore, algorithms were only considered if they were able to extract gait (sequences) or strides that could be assembled to GSs as previously described [11,40], that is, strides were only combined to a GS if they were not further apart than 3 seconds.

Wherever possible, algorithm parameters were optimized as follows. First, it was deemed necessary to replace any axis-specific dependency by the 3D accelerometer signal norm, if possible. While the lower back provides a rather constant vertical orientation with respect to the global world coordinate system during walking, the axis orientations of wrist sensors change constantly due to free arm movement. Using the norm as orientation-independent signal was the most natural choice without introducing any other sensor-body alignment process. Second, algorithm-specific parameters were optimized on the *optimization data set* of 11 young and healthy participants (described earlier). A grid search was used to assess algorithm performance for different algorithm parameter combinations on the *optimization data set*. The best-performing parameter combination was used for further validation of the algorithms on the *validation data set* (participants from all the 6 different participant groups). Algorithm performance was evaluated as described below. The Paraschiv-Ionescu (2020) algorithm was not optimized as it contains a data-adaptive threshold. The Brand (2020) algorithm was initially developed specifically for analyzing data from wrist-worn inertial sensors. Consequently, the algorithm remained largely unchanged, except for modifying the training data. Thus, only the optimized version, which involved training the model on the *optimization data set*, was evaluated.

The performance of the wrist-based algorithms was compared to 3 lower back–based algorithms, which have previously been validated on the same data set [11]. This includes the algorithms Iluz (2014) (denoted GSD_A_ previously [11]) and Paraschiv-Ionescu (2019) (denoted GSD_B_ and GSD_C_ previously [11]). The specific lower back algorithm parameters for the lower back are described in Table 1.

**Table 1:** Algorithm descriptions including overview over default and tuned algorithm parameters. GSDA^a^, GSDB, and GSDC are algorithm versions described and validated for the lower back [11].

Domain	Algorithm name (reference)	Description	Original sensor position	Algorithm parameters (default)	Algorithm parameters (optimized)
Machine learning^b^	Brand (2022) [17]	Use of deep convolutional neural network to discriminate gait and nongait segments based on accelerometer data.	Wrist	N/A^c^	CNN^d^ trained on Mobilise-D optimization data set (see Methods)
Time domain^e^	Gu (2017) [41,42]	This method finds peaks in the summed and squared (RMS^f^) acceleration signal. It uses multiple thresholds to determine if each peak belongs to a step or artifact.	Wrist	verisense_k=3 sim_thres=–0.5 cont_thres=4 mag_thres=1.2	verisense_k=2 sim_thres=–0.8 cont_thres=4 mag_thres=1.2
Time domain^g^	Hickey (2017) [43]	Window-based threshold comparison of combined SD of 3D acceleration signal and vertical acceleration.	Lower back	ThresholdStill=0.2 ThresholdUpright=–0.5	ThresholdStill=0.2 ThresholdUpright=–0.5
Template based^g^	Iluz (2014) (GSD_A_) [44]	Convolution of input signal with a gait cycle template (sine wave). Detection of local maxima in convolution result to define regions of gait.	Lower back	Vertical and anteroposterior acceleration used (lower back) activity_thres=0.01 min_bout_length=5 template_len=0.5 cm_norm_thres=0.4	Vertical and anteroposterior acceleration replaced by acceleration norm activity_thres=0.04 min_bout_length=10 template_len=1 cm_norm_thres=2.5
Template based^e^	Karas (2019) [31]	Template-based method (considering covariance between a scaled and translated pattern function) for stride detection based on adaptive empirical pattern transformation.	Wrist	sim_MIN=0.85 dur_MIN=0.8 dur_MAX=1.4 ptp_r_MIN=0.2 ptp_r_MAX=2.0 mean_abs_diff_med_p_MAX=0.5 mean_abs_diff_med_t_MAX=0.2 mean_abs_diff_dur_MAX=0.2	sim_MIN=0.3 dur_MIN=0.2 dur_MAX=3.0 ptp_r_MIN=0.2 ptp_r_MAX=3.0 mean_abs_diff_med_p_MAX=0.5 mean_abs_diff_med_t_MAX=0.5 mean_abs_diff_dur_MAX=0.5
Time domain^b^	Kheirkhahan (2017) [45]	Based on ActiGraph activity counts using sliding windows and adaptive thresholds.	Lower back	Walking threshold=0.75	Walking threshold=0.6
Time domain^g^	Paraschiv-Ionescu (2019) (GSD_B_ and GSD_C_) [46]	Locomotion period detection based on detected steps from the Euclidean norm of the accelerometer signal. Consecutive steps are associated to gait sequences.	Lower back	GSDB: th=0.1 GSDC: th=0.15	Wrist: th=0.35
Time domain^g^	Paraschiv-Ionescu (2020) [47]	Extension of Paraschiv-Ionescu (2019). It applies an improved preprocessing strategy for the acceleration norm including an iterative succession of smoothing and enhancement stages. Furthermore, a data-adaptive threshold was introduced.	Lower back	N/A	N/A
Frequency domain^g^	Wavelets (Proprietary, Center for the Study of Movement, Cognition, and Mobility. Tel Aviv Sourasky Medical Center, Tel Aviv, Israel)	Time-frequency analysis using wavelets.	Lower back	Vertical and anterio-posterior acceleration used (lower back)	Vertical acceleration replaced by acceleration norm
Machine learning^b^	Willetts (2018) [20]	Activity detection using random forests and hidden Markov models to detect various activity modes. Only the output for “walking” activity was considered.	Wrist	Epoch length: 30 seconds	Epoch length: 1 second

^a^GSD: gait sequence detection.

^b^Programming language is Python (Python Software Foundation).

^c^N/A: not applicable.

^d^CNN: convolutional neural network.

^e^Programming language is R (R Foundation for Statistical Computing).

^f^RMS: root-mean-square.

^g^Programming language is Matlab (MathWorks).

### Gait Detection Validation Metrics Evaluation

The output of the gait detection algorithms yielded start and end times for all GSs. Each 2.5-hour recording (containing a varying number of GSs) was segmented into windows of 0.1 seconds as previously described [11]. Based on the comparison of the algorithm output to the reference system, each window was classified as true positive (TP), false positive (FP), true negative (TN), or false negative (FN) regarding the detection of gait [11]. For each 2.5-hour recording, the following metrics were calculated:

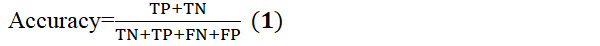

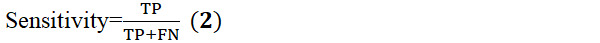

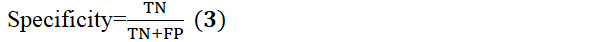

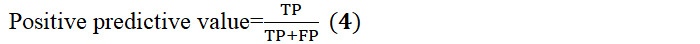









Furthermore, errors of the total duration of all GSs and for the number of detected GSs in each 2.5-hour recording were calculated. The relative and relative absolute errors were determined as a ratio between the (absolute) errors per GS and the corresponding estimates from the reference system, expressed as a percentage. All metrics were calculated for each participant and for the algorithms applied to both wrist sensor versus reference system as well as to lower back sensor versus reference system. We aggregated the error metrics on a disease group level using the mean.

The intraclass correlation coefficient (ICC_2,1_) [48] was calculated for the total GS duration for each 2.5-hour recording on a participant group level (n=6). Values smaller than 0.50, between 0.50 and 0.75, between 0.75 and 0.90, and larger than 0.90 were indicative of poor, moderate, good, and excellent reliability, respectively [49].

A previously described methodology to combine the above metrics into 1 performance index ranging between 0 (worst) and 1 (best) was used [50]. This index is calculated based on a weighted combination of the above-defined metrics (accuracy, sensitivity, specificity, sensitivity, positive predictive value, ICC, mean GS duration relative absolute error, and mean GS number relative absolute error). Each metric can be considered a cost or benefit metric contributing to the performance index with a specific weight (Multimedia Appendix 1). This enables a direct comparison and ranking of the algorithm performances [11]. The performance index was calculated per disease group (n=6).

### Statistical Comparison of Algorithm Performance (Wrist vs Lower Back)

For each algorithm, the optimized version (if available) was compared against a representative algorithm for the lower back [Iluz (2014)] with a 2-sided paired *t* test on a participant level for each performance metric and adjusted *P* values for multiple testing using Benjamini and Hochberg procedure [51].

### Influence of Walking Aids on Algorithm Performance

As the *validation data set* includes participants with a potential need to use walking aids during the assessment, the effect of walking aids was investigated on algorithm performance. Information was available about (1) whether a walking aid was used and (2) what type of walking aid (among 1-sided canes or crutches, 2 crutches, rollators, and walkers) was used during the 2.5-hour free-living recording.

## Results

### Population Overview

Of the 108 recruited participants, 25 participants were excluded from subsequent analysis, as either reference, wrist, or lower back sensor data were missing or incomplete (HA: n=3, MS: n=7, Parkinson disease: n=5, PFF: n=8, and CHF: n=2). Wrist and lower back validation were based on the same set of participants. Thus, 83 participants were included in the validation analysis. Overall, 10 participants used walking aids. Participants’ clinical and demographic characteristics per disease group are shown in Table 2.

**Table 2:** Demographic and clinical characteristics of the participants included in the real-world analysis. The gait sequence (GS) information is based on the GSs detected by the reference system given per 2.5-hour recording. Gait duration is given as sum over all GSs in one 2.5-hour recording.

Characteristics	Validation sample							Optimization sample
	HA^a^	CHF^b^	COPD^c^	MS^d^	PD^e^	PFF^f^	HA	
Participants, n (%)	17 (18.1)	10 (10.6)	17 (18.1)	13 (13.8)	15 (16)	11 (11.7)	11 (11.7)	
Age (years), mean (SD)	72.35 (6.00)	68.60 (12.21)	69.35 (9.10)	47.23 (11.09)	69.20 (7.48)	79.70 (6.86)	29.55 (7.76)	
Height (cm), mean (SD)	167.00 (10.91)	174.40 (10.27)	168.97 (6.61)	166.31 (9.11)	172.73 (7.96)	170.23 (9.07)	174.64 (9.24)	
Weight (kg), mean (SD)	74.36 (12.53)	83.75 (18.44)	73.71 (14.22)	80.09 (22.11)	79.13 (16.27)	70.59 (16.86)	69.09 (11.35)	
Walking aid users, n (%)	0 (0)	4 (40)	0 (0)	3 (23)	1 (7)	2 (18)	0 (0)	
Walking aid types	—^g^	One cane or crutch: 2; rollator: 2	—	One cane or crutch: 1; 2 crutches: 1; walker: 1	Rollator: 1	One cane or crutch: 2	—	
MoCA^h^ (0-30), mean (SD)	28.18 (1.38)	26.70 (3.06)	24.65 (3.39)	26.23 (3.49)	23.93 (4.45)	25.09 (4.46)	—	
Hoehn and Yahr stage, n	N/A^i^	N/A	N/A	N/A	I: 3, II: 7, III: 5	N/A	N/A	
MDS-UPDRS III^j^ (0-132), mean (SD)	N/A	N/A	N/A	N/A	30.67 (13.33)	N/A	N/A	
EDSS^k^ (0-6), mean (SD)	N/A	N/A	N/A	3.85 (1.72)	N/A	N/A	N/A	
SPPB^l^ (0-12), mean (SD)	N/A	N/A	N/A	N/A	N/A	7.73 (3.10)	N/A	
CAT^m^ score (0-40), mean (SD)	N/A	N/A	19.65 (8.95)	N/A	N/A	N/A	N/A	
FEV_1_^n^ (L), mean (SD)	N/A	N/A	1.58 (0.58)	N/A	N/A	N/A	N/A	
6MWT^o^ distance (m), mean (SD)	N/A	323.50 (171.46)	357.65 (88.52)	N/A	N/A	N/A	N/A	
Gait duration (minutes), median (IQR)	27.2 (25.5-30.1)	17.8 (9.9-29.7)	17.2 (12.9-21.1)	12.3 (9.4-18.3)	13.9 (10.9-24.7)	19.2 (13.0-24.8)	39.2 (36.4-64.2)	
Number GS, median (IQR)	66 (56-88)	55 (21-71.8)	71 (37-80)	37 (18-45)	31 (22-46)	37 (30.5-51)	36 (23-42.5)	

^a^HA: healthy older adult.

^b^CHF: congestive heart failure.

^c^COPD: chronic obstructive pulmonary disease.

^d^MS: multiple sclerosis.

^e^PD: Parkinson disease.

^f^PFF: proximal femoral fracture.

^g^Not available.

^h^MoCA: Montreal Cognitive Assessment.

^i^N/A: not applicable.

^j^MDS-UPDRS III: Movement Disorder Society Unified Parkinson Disease Rating Scale Part III.

^k^EDSS: Expanded Disability Status Scale.

^l^SPPB: short physical performance battery.

^m^CAT: Chronic Obstructive Pulmonary Disease Assessment Test.

^n^FEV_1_: forced expiratory volume in 1 second.

^o^6MWT: 6-minute walking test.

### Algorithms

Overall, 10 algorithms were included in this validation study (Table 1). They included algorithms originally used for the lower back as well as wrist-specific algorithms. They can be grouped into different domains: (1) time- or frequency domain–based, (2) stride template–based, and (3) machine learning algorithms. All implemented algorithms have been adapted to use the 3D accelerometer signal only. Algorithm-specific parameters were optimized on the *optimization data set* (Table 1).

### Performance Results

**Figure 1:**
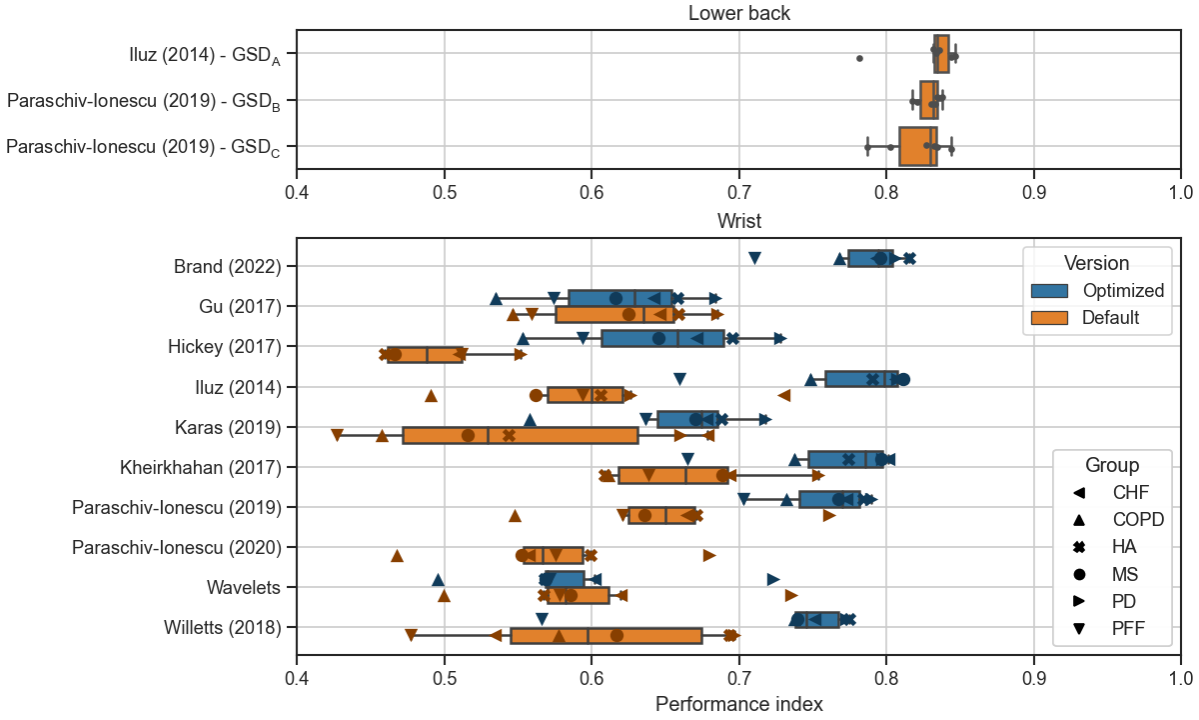
Performance of assessed algorithms based on a disease group level (n=6). Individual data points are highlighted for each disease group as an overlay. The names of lower back algorithms are given as defined previously [11] and referred to in Table 1. Boxes indicate lower and upper quartiles; the whiskers correspond to 1.5 IQR. Colors indicate the algorithm version: orange indicates the default algorithm version without optimized parameters, and blue indicates the optimized algorithm (parameter tuning based on the optimization data set). In the “wrist” subplot, shapes indicate the disease group to visualize algorithm performance for each group. CHF: congestive heart failure; COPD: chronic obstructive pulmonary disease; GSD: gait sequence detection; HA: healthy older adults; MS: multiple sclerosis; PD: Parkinson disease; PFF: proximal femoral fracture (hip fracture recovery).

The performance of most optimized algorithms increased compared to using default algorithm parameters (Figure 1). The optimized versions of the Brand (2022) and Paraschiv-Ionescu (2019) algorithms had a performance index above 0.7 for all groups, with the Brand (2022) algorithm showing the highest performance (Figure 1). In the following, the wrist results for the optimized algorithm versions are reported.

Regarding wrist-based GSD, the performance index of the algorithms Willetts (2018), Iluz (2014), and Kheirkhahan (2017) was between 0.74 and 0.81 for most disease groups, except for the PFF group (Multimedia Appendix 2). In the PFF group, the performance index was 0.66 for the Iluz (2014) and Kheirkhahan (2017) algorithms, while it was 0.57 for the Willetts (2018) algorithm.

**Figure 2:**
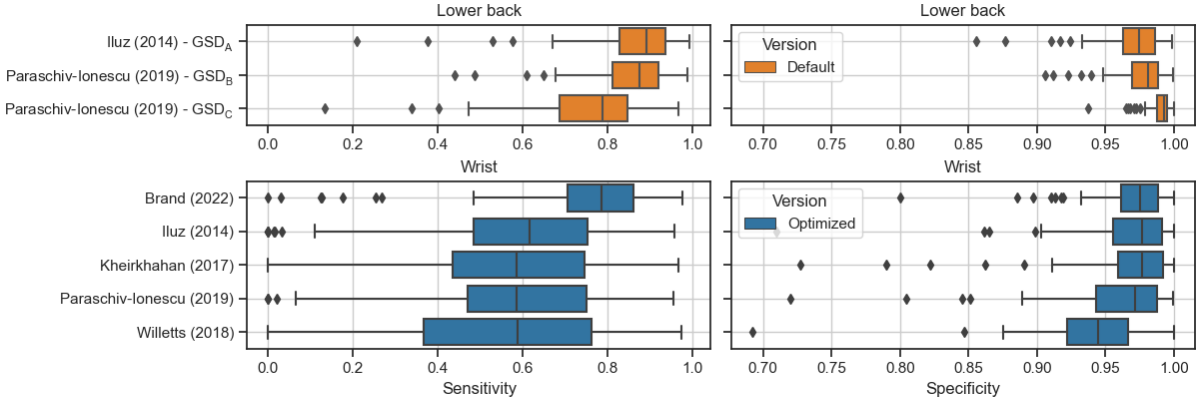
Sensitivity (left) and specificity (right) for the best-performing wrist algorithms (performance index higher than 0.7 for most disease groups except proximal femoral fracture) based on a participant level (N=83). Colors indicate the algorithm version: orange indicates the default algorithm version without optimized parameters, and blue indicates the optimized algorithm with parameter tuning based on the optimization data set. GSD: gait sequence detection.

For the 5 best-performing algorithms Brand (2022), Paraschiv-Ionescu (2019), Iluz (2014), Kheirkhahan (2017), and Willetts (2018), the mean sensitivity ranged between 0.52 (SD 0.28) and 0.81 (SD 0.09) (when excluding the PFF group), whereas the only algorithm showing mean sensitivity (per disease group) consistently higher than 0.70 was Brand (2022). The specificity for those algorithms was between 0.91 and 0.98. ICC values (for GS duration) ranged between 0.72 and 0.99. For PFF, the performance was consistently lower, with sensitivity ranging between 0.29 and 0.55, specificity between 0.94 and 0.96, and ICC values between 0.08 and 0.83 (Figure 2 and Multimedia Appendix 2).

**Figure 3:**
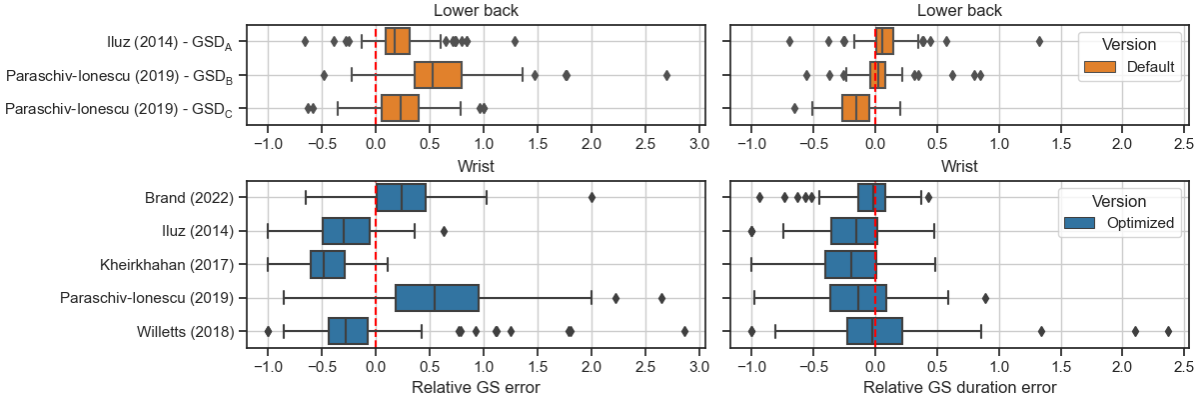
Relative errors of the estimated number of GSs (left) and of the estimated gait duration (right) per 2.5-hour recording based on a participant level (N=83). The dashed red line represents an error of 0 (optimal result). Negative relative errors indicate that fewer GS were detected or the total GS duration was lower than estimated by the reference system. The figure includes the best-performing algorithms (performance index higher than 0.7 for most disease groups except proximal femoral fracture). Colors indicate the algorithm version: orange indicates the default algorithm version without optimized parameters, and blue indicates the optimized algorithm with parameter tuning based on the optimization data set. GS: gait sequence; GSD: gait sequence detection.

The mean relative absolute error of the total estimated gait duration during the 2.5-hour recordings was between 8.9% (SD 7.1) (HA) and 32.7% (SD 19.2) (PFF) for the best-performing algorithm, that is, Brand (2022). The Paraschiv-Ionescu (2019) algorithm showed an error between 22% (HA) and 38% (PFF), while the other algorithms performed worse. The mean relative absolute error regarding the number of detected GSs in the 2.5-hour recording ranged between 22.3% (SD 21.1) (HA) and 44.6% (SD 55.3) (PFF) for the Brand (2022) algorithm and worse for the other algorithms (Multimedia Appendix 2). Figure 3 visualizes the relative errors indicating whether the algorithms under- or overestimate the number and duration of detected GSs.

For the reported algorithms applied to the lower back position, sensitivity ranged between 0.71 and 0.91, specificity between 0.96 and 0.99, and ICC values between 0.68 and 1.0 (Multimedia Appendix 3). Overall, algorithms applied to wrist signals resulted in lower performance compared to the lower back position as shown in Figure 1 and quantified as follows. Differences in validation metrics of algorithms applied to either wrist compared to the lower back algorithm Iluz (2014) were statistically assessed (Table 3) based on the validation metrics per participant (Multimedia Appendix 4). For sensitivity, all algorithms for the wrist are different (*P*<.001) from GSD_A_, with the Brand (2022) algorithm having the smallest difference in mean (–0.126), and Willetts (2018) the largest (–0.317) compared to the lower back algorithm Iluz (2014). For specificity, the Brand (2022), Iluz (2014), and Kheirkhahan (2017) algorithms are not significantly different (*P*>.10) from GSD_A_, with the Brand (2022) algorithm having the smallest difference (–0.00192). The Brand (2022) algorithm is closest to GSD_A_ for relative error in number of detected GSs (*P*=.021 and a difference in mean of 0.042). No statistical comparison was conducted for the performance index itself, as the index was calculated only per disease group, resulting in a small sample of 6 data points.

**Table 3:** Statistical results comparing each wrist algorithm and metric (optimized versions) with a representative lower back algorithm with good performance (Iluz (2014) applied to the lower back).

Algorithm and metric			Mean difference		*P* value	Adjusted *P* value
**Willetts (2018)**						
	Sensitivity	–0.317		<.001		<.001
	Specificity	–0.029		<.001		<.001
	PPV^a^	–0.258		<.001		<.001
	Accuracy	–0.064		<.001		<.001
	Relative number GS^b^ error	–0.325		<.001		<.001
	Relative GS duration error	–0.055		.37		.38
**Brand (2022)**						
	Sensitivity	–0.126		<.001		<.001
	Specificity	–0.002		.49		.49
	PPV	–0.037		.03		.04
	Accuracy	–0.014		<.001		<.001
	Relative number GS error	0.042		.19		.21
	Relative GS duration error	–0.131		<.001		<.001
**Paraschiv-Ionescu (2019)**						
	Sensitivity	–0.277		<.001		<.001
	Specificity	–0.011		<.001		<.001
	PPV	–0.115		<.001		<.001
	Accuracy	–0.041		<.001		<.001
	Relative number GS error	0.335		<.001		<.001
	Relative GS duration error	–0.216		<.001		<.001
**Iluz (2014)**						
	Sensitivity	–0.273		<.001		<.001
	Specificity	–0.004		.27		.29
	PPV	–0.067		<.001		<.001
	Accuracy	–0.035		<.001		<.001
	Relative number GS error	–0.500		<.001		<.001
	Relative GS duration error	–0.268		<.001		<.001
**Kheirkhahan (2017)**						
	Sensitivity	–0.299		<.001		<.001
	Specificity	–0.005		.23		.25
	PPV	–0.063		<.001		<.001
	Accuracy	–0.038		<.001		<.001
	Relative number GS error	–0.671		<.001		<.001
	Relative GS duration error	–0.310		<.001		<.001

^a^PPV: positive predictive value.

^b^GS: gait sequence.

### Effect of Walking Aids

**Figure 4:**
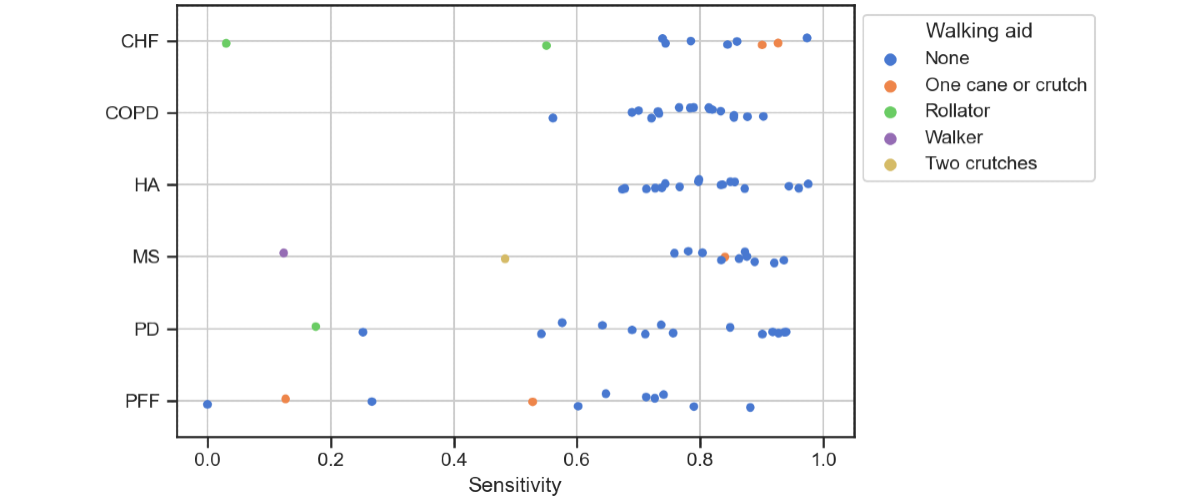
Effect of walking aids on sensitivity for the algorithm Brand (2022) [17]. Each data point represents 1 participant (N=83), the color indicates the type of walking aid. CHF: congestive heart failure; COPD: chronic obstructive pulmonary disease; HA: healthy older adults; MS: multiple sclerosis; PD: Parkinson disease; PFF: proximal femoral fracture (hip fracture recovery).

The frequency of walking aid use depended on the disease group (Table 2). Walking aid use influenced the accuracy of wrist-based gait detection (Figure 4). Participants using bilateral walking aids (rollators, walkers, and 2 crutches) exhibited lower sensitivity for gait detection. The gait of 5 participants using unilateral walking aids (CHF: n=2, MS: n=1, and PFF: n=2) was not as affected and could mostly be estimated as accurately as for participants without walking aids. For the unilaterally used walking aids (1 cane or crutch), 2 of 5 participants wore the sensor on the same side as they used the walking aid. An exception was the PFF group, in which low sensitivity has also been observed for unilateral walking aid use. In total, 3 patients using no walking aid showed a sensitivity below 0.5, and 2 of those participants reported to usually use walking aids (but not in this study), while the third participant showed a low short physical performance battery score of 4, indicating that walking might be strongly impaired.

## Discussion

### Principal Findings

This is the most comprehensive study so far, evaluating real-world gait detection performance of various algorithms from a wrist-worn sensor in a heterogenous population including 5 disease cohorts.

### Performance Results

The Brand (2022) and Paraschiv-Ionescu (2019) algorithms exhibited good performance (>0.75) across all disease groups excluding PFF (moderate performance index of 0.71), while the first outperformed the latter algorithm especially for the total estimated walking time and the number of detected GSs. The Iluz (2014) and Kheirkhahan (2017) algorithms also showed high performance, except for the PFF group (performance index of 0.66). A lower performance was generally observed for the PFF group. This has already been previously reported for the lower back position [11] and can be attributed to several factors, which significantly impacts the accuracy of gait detection algorithms. First, patients with PFF may show altered gait patterns due to pain, muscle weakness, and impaired mobility. Second, they may exhibit asymmetrical walking, making it harder for algorithms to identify consistent patterns. Finally, the gait of hip fracture recovery patients may vary more widely even within the same group, which is also reflected, for example, in the range of number of GSs, which was highest for the PFF group in this study (Table 2).

Based on those results, we suggest the use of the Brand (2022) algorithm, which is suitable for gait detection based on wrist-worn sensors across all investigated disease groups.

Sensitivity and specificity were calculated with regard to the agreement of gait detection algorithm results to the reference GSs based on 0.1-second windows for complete 2.5-hour recordings. Sensitivity was generally lower than specificity, indicating that not all GSs were detected by all algorithms. On the other hand, high specificity indicates that only few nongait activities are misclassified as GSs. Further algorithm optimization on a larger data set is required to find the optimal balance between sensitivity and specificity. If the goal is to subsequently characterize DMOs, a high specificity is needed to exclude nonwalking periods (including transitions and shuffling of gait), but at the same time accept a portion of missed walking periods.

### Comparison to Lower Back

Due to the high movement variability of the arm during walking, a performance drop is expected when comparing algorithms applied to a wrist-worn versus a lower back–worn inertial sensor. This performance drop is evident in this study. Sensitivity is lower for the wrist position (sensitivity was between 0.32 and 0.13 smaller compared to (Iluz 2014) applied on lower back data, Table 3), which can most likely be attributed to nonperiodical arm swing with differences in amplitude during walking. However, specificity is comparably high (>0.7) for both sensor positions (Figure 2), indicating the general reliability of correctly rejecting nongait activities.

### Algorithm Parameter Optimization

The design of some of the algorithms focused on the lower back position initially (Table 1). However, in this study, we focused on implementing methods initially developed for lower back acceleration signals based on time or frequency methods to wrist acceleration signals. Where possible, algorithm parameters were optimized on the *optimization data set*. Default and optimized algorithm parameters differed, and optimization allowed for achieving higher performances of lower back algorithms at the wrist position.

A strong advantage of all investigated algorithms is that they use the 3D accelerometer signal only and do not depend on gyroscopic sensors, sensors that have high energy consumption. Thus, they can potentially be used more ubiquitously for other wearable inertial sensors that acquire accelerometer data only, allowing for energy-efficient gait detection systems and, thus, longer assessment periods. In addition, in future work, we will evaluate the effect of lower accelerometer sampling rates (eg, 30 Hz instead of 100 Hz) on GSD performance. The use of inexpensive, low-sampling consumer-grade watches in public health projects may justify reduced performance as observed in this study.

### Walking Aids

Walking aid users move differently due to several factors. Gait impairment can be observed in various diseases including the groups assessed within this study; in addition, the use of walking aids may lead to compensatory gait changes and can influence gait parameters directly [52,53].

Furthermore, biomechanical constraints when using walking aids affect wrist-based gait assessment. Bilateral walking aids such as walkers or rollators may significantly affect the arm movement and thus the acquired accelerometer signals. On the one hand, this can be used to construct specific algorithms for gait assessment when walking aids are used [54]. This, in turn, may lead to deteriorated performance of algorithms that are not fit for the purpose of walking aid–based gait assessment.

The results of this study demonstrate that special care must be taken when defining inclusion and exclusion criteria in studies based on a wrist-worn sensor for gait assessment. Participants using rollators, walkers, or 2 crutches may be separately considered in wrist-based gait assessment. However, the actual use of walking aids in real-world environments can hardly be predicted. Unilateral walking aids can potentially be used on either side and switched during the assessment. Participants may also not use walking aids continuously but only when they feel unsafe (depending on the environment) and may also use other everyday objects (eg, furniture) for increased security, which may affect the interpretation of sedentary or activity levels.

### Strengths and Limitations

The focus of this study was to investigate the performance of gait detection algorithms on real-world data, in which full reference information from the sensor-based INDIP system including pressurized insoles was available. We see the use of this multimodal reference system as a unique advantage compared to data sets used in previous studies, as it allows not only to assess gait detection very accurately but also to extract other spatiotemporal gait parameters. The accuracy of this system has previously been assessed against an optical motion capture system and has showed excellent absolute agreement (ICC>0.95) within a laboratory setting [34]. We thus considered the INDIP system as a reliable method for acquiring reference data in real-world environments. One can argue that the 2.5-hour assessment used for validation might not fully represent the full variability of real-world walking. Nevertheless, our data set is one of the largest available ones containing full reference information for a variety of disease indications. Future work could use longer validation periods. Overall, a diverse set of disease areas were represented including orthopedic, pulmonary, cardiovascular, and neurological diseases. Future studies could extend this work to other disease groups.

It is worth noting that the optimization set used for this study was relatively small and comprised a population that differed from the validation set in terms of age and health condition. It was based on a healthy young adult group that did not rely on the use of walking aids, which might bias the optimal parameter choice. Algorithm performance could likely be improved using disease-specific samples including walking aid users or tuning even based on individual participants. Future studies should, thus, focus on optimizing gait detection algorithms specifically tailored for participants with gait disturbances related to the disease groups of interest. The methodology of this paper can serve as a reference for achieving this.

Real-world data are naturally imbalanced, with a significantly larger number of nongait segments (majority class) compared to gait segments (minority class). This inherent imbalance can introduce bias in supervised models, resulting in low sensitivity. Consequently, further analyses should focus on addressing this problem by using techniques such as upsampling from the minority class or generating artificial samples.

We acknowledge that the list of included algorithms might not be exhaustive. Our choice was driven by practical considerations including code availability and applicability on wrist-worn accelerometer data. However, the algorithms cover a broad spectrum of different domains using time and frequency domain, template matching, and machine learning methods. Future work may compare further, also proprietary, algorithms to the presented results. In addition, the Mobilise-D technical validation data set as well as the used validation methodology might provide a blueprint for future validation studies.

This paper only validated the first step of a complete gait analysis pipeline. Future work will need to show whether subsequently extracted DMOs such as cadence, stride length, and walking speed can reliably be estimated for those identified GSs in comparison to the lower back.

### Conclusions

To conclude, we identified algorithms that can extract GSs based on a wrist-worn sensor using accelerometer data. In general, the performance for detecting GSs as regions of interest of further gait parameter extraction and quantification of gait duration is lower than for the lower back position. However, the omnipresence of wrist-worn sensors and their easier operationalization and better ergonomics in longitudinal clinical trials may justify some level of lower gait quantification performance for the sake of higher acceptance and more data. Identifying GSs in continuous long-term inertial sensor recordings is the first step that will allow extracting additional DMOs (eg, spatiotemporal parameters such as walking speed in disease cohorts [21,22]).

Our work is a step toward quantifying the limitations of wrist-worn devices for digital mobility analysis and contributes to the evidence needed by researchers, clinical trial teams, and health care professionals in deciding if a lower back inertial sensor is required or a wrist-worn sensor is sufficient. The data presented here should be considered as one part of further opportunities offered by wrist-worn inertial sensors. To assess a comprehensive movement picture of patients, different algorithms can, for example, measure further DMOs related to mobility analysis, including spatiotemporal parameters and physical activity. Quantification of continuous GSs may be a DMO on its own that can be explored in diseases with reduced physical performance.

## Appendix

Multimedia Appendix 1 Weights used for calculating the performance index.

Multimedia Appendix 2 Gait sequence detection performance metrics and overall performance index for the wrist sensor position (optimized versions, if available). Values are provided as mean and [5%, 95%] quantiles or as mean and limit of agreement (LoA).

Multimedia Appendix 3 Gait sequence detection performance metrics and overall performance index for the lower back sensor position. Values are provided as mean and [5%, 95%] quantiles or as mean and limit of agreement.

Multimedia Appendix 4 Gait sequence detection metrics for each 2.5 h recording per sensor position, algorithm, and participant.

## Abbreviations

CHF: congestive heart failure
DMO: digital mobility outcome
FN: false negative
FP: false positive
GS: gait sequence
GSD: gait sequence detection
HA: healthy older adult
ICC: intraclass correlation coefficient
INDIP: inertial module with distance sensors and pressure insoles
MS: multiple sclerosis
PFF: proximal femoral fracture
TN: true negative
TP: true positive

## Data Availability

Example data sets generated and analyzed during this study are available in the Zenodo repository [55]. The full data set will be made available by the Mobilise-D consortium by June 2024.
